# Aromatase inhibitors: the journey from the state of the art to clinical open questions

**DOI:** 10.3389/fonc.2023.1249160

**Published:** 2023-12-22

**Authors:** Daniele Generali, Rossana Berardi, Michele Caruso, Marina Cazzaniga, Ornella Garrone, Ida Minchella, Ida Paris, Carmine Pinto, Sabino De Placido

**Affiliations:** ^1^ Breast Cancer Unit, Azienda Socio Sanitaria Territoriale di Cremona, Cremona, Italy; ^2^ Department of Medical, Surgery and Health Sciences, University of Trieste, Trieste, Italy; ^3^ Medical Oncology, Azienda Ospedaliera Universitaria (AOU) delle Marche, University Politecnica delle Marche, Ancona, Italy; ^4^ Humanitas Istituto Clinico Catanese, Breast Centre Humanitas Catania, Catania, Italy; ^5^ School of Medicine and Surgery University of Milano Bicocca, Milan, Italy; ^6^ Phase 1 Research Unit, Azienda Socio Sanitaria Territoriale (ASST) Monza, Monza, Italy; ^7^ Medical Oncology, Fondazione IRCCS Ca’ Granda Ospedale Maggiore Policlinico, Milan, Italy; ^8^ Division of Early Drug Development, European Institute of Oncology IRCCS, Milan, Italy; ^9^ Department of Woman and Child Health and Public Health, Division of Gynecologic Oncology, Fondazione Policlinico Universitario Agostino Gemelli Istituto di Ricerca e Cura a Carattere Scientifico (IRCCS), Rome, Italy; ^10^ Medical Oncology Unit, Comprehensive Cancer Centre, Azienda Unità Sanitaria Locale - Istituto di Ricerca e Cura a Carattere Scientifico (AUSL-IRCCS) di Reggio Emilia, Reggio Emilia, Italy; ^11^ Department of Clinical Medicine and Surgery, University of Naples Federico II, Naples, Italy

**Keywords:** aromatase inhibitors, bone loss, cardiotoxicity, drug adherence, breast cancer

## Abstract

Breast cancer is a major cause of death among females. Great advances have been made in treating this disease, and aromatase inhibitors (AIs) have been recognized as the cornerstone. They are characterized by high efficacy and low toxicity. The authors reviewed the available literature and defined state-of-the-art AI management. This study was designed to assist clinicians in addressing the need to equally weigh patients’ needs and disease control rates in their everyday clinical practice. Today, AIs play a central role in the treatment of hormone receptor-positive breast cancer. In this study, an expert panel reviewed the literature on the use of AIs, discussing the evolution of their use in various aspects of breast cancer, from pre- and postmenopausal early breast cancer to metastatic breast cancer, along with their management regarding efficacy and toxicity. Given the brilliant results that have been achieved in improving survival in everyday clinical practice, clinicians need to address their concerns about therapy duration and the adverse effects they exert on bone health, the cardiovascular system, and metabolism. Currently, in addition to cancer treatment, patient engagement is crucial for improving adherence to therapy and supporting patients’ quality of life, especially in a selected subset of patients, such as those receiving an extended adjuvant or combination with targeted therapies. A description of modern technologies that contribute to this important goal is provided.

## 1 Introduction

In 2018, GLOBOCAN showed that breast cancer accounted for approximately 2.1 million diagnoses and 630,000 deaths in 185 countries (1). In 2020, the same source reported a higher incidence of the disease, accounting for approximately 2.3 million diagnoses and 690,000 deaths worldwide (2).

A perfect balance between the various approaches should be achieved when treating breast cancer. Surgery, neoadjuvant and adjuvant chemotherapy, endocrine therapy, targeted therapy, and other methods are available in the therapeutic armamentarium of modern oncologists. Specifically, endocrine therapy with selective estrogen receptor (ER) modulators (SERMs), such as tamoxifen (TAM) and aromatase inhibitors (AIs), is currently central to the treatment of hormone receptor-positive breast cancer (3–6).

To describe the “state of the art” on using AIs, the panel searched Pubmed with the keywords “breast cancer, aromatase inhibitors, bone loss, cardiotoxicity, drug adherence.” The authors believe that it is mandatory to include meta-analyses, reviews, systematic reviews, and randomized controlled trials published in the last 15 years, focusing on the results achieved with the use of AIs with particular attention to their toxicity profiles and keeping an eye on the management of patients undergoing extended AI therapy.

## 2 History of aromatase inhibitors

The first report on the use of an antiestrogenic drug in patients with advanced breast cancer was published in 1971 (7). This approach was first approved in the United Kingdom (1973) and later in the United States (1977). TAM was, at that time, the first endocrine agent named SERM, which was able to function as an antagonist of the ER in breast cancers. Aminoglutethimide is a nonselective AI used in postmenopausal patients, which demonstrated the ability to achieve a 90% reduction in circulating estrogen levels. Its clinical use was approved in 1980; however, it did not show any difference regarding efficacy compared with TAM in randomized controlled trials (8–10). Second-generation AIs (such as formestane, fadrozole, and vorozole) were intended to overcome aminoglutethimide side effects, leading to the development of drugs such as 4-hydroxyandrostenedione; however, they still lack selectivity (11). The third and last generation of AIs includes both steroidal (exemestane) and nonsteroidal (anastrozole and letrozole) drugs, with greater specificity and fewer side effects (12).

The exemestane, letrozole, and anastrozole mechanisms of action reside in the inhibition of the aromatase enzyme, which can drive the conversion of androgens into estrogens through the “aromatization pathway.” In postmenopausal women, the breast tissue is enhanced by the intratumoral production of estrogens. The inhibition of estrogen conversion is one of the main ways to suppress breast cancer relapses (13).

The drugs are considered long-acting based on their pharmacokinetic dosing interval, as their half-life is estimated to last over 42 h in patients with breast cancer (13, 14). They block the CYP19A1 chain by inhibiting its active site, resulting in loss of electron transfer. This blockade prevents the conversion of androgens into estrogens (15). Moreover, this class of drugs has negligible effects on blood cortisol, aldosterone, and thyroxine blood levels (13). All are oral and have been approved by the regulatory agencies active in each country for treating breast cancer in routine clinical practice.

## 3 AIs: a landmark in ER-positive breast cancer

**Figure 1:**
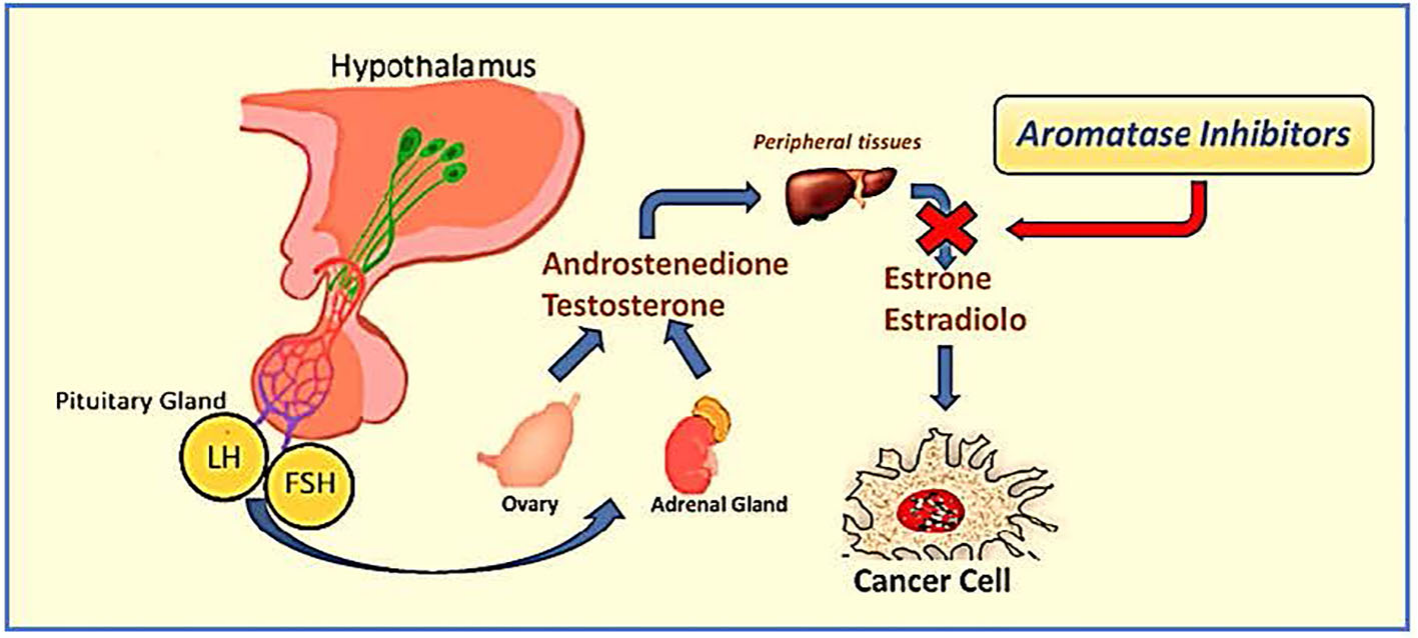
Mechanism and level of action of aromatase inhibitors.

Historically, patients with ER-positive breast cancer treated with TAM for 5 years had a decreased risk of death by approximately half during pharmacological treatment. This risk increases to about one-third at 15 years (16). AIs impede the conversion of androgens to estrogens; therefore, they cannot be adopted in premenopausal women unless they are exposed to ovarian function suppression (OFS) (17). In contrast, in postmenopausal women, AIs reduce the serum estrogen levels, thereby inhibiting ER-positive breast cancer cell stimulation (**Figure 1**).

The literature offers various trials that compare AIs and TAM in adjuvant endocrine therapy (AET) (18–26). The Early Breast Cancer Trialists’ Collaborative Group (EBCTCG) meta-analysis was released in 2015 to clarify the relative benefits of AIs vs. TAM and the outcomes of scheduling these two drugs differently during the 5 years of AET (27). The authors included data from nine trials with 35,129 females enrolled and randomized to AIs and TAM. During this meta-analysis, all the trials had already demonstrated the ability of AIs to lower recurrence rates when compared with TAM. Thus, this paper aims to establish AIs’ role in decreasing breast cancer and all-cause mortality. The authors observed that 5 years of endocrine therapy with AIs can lessen breast cancer relapses by about one-third during years 5 to 9, as does 5 years of TAM. Moreover, when comparing TAM given for 2 years followed by AIs with TAM given for 2–4 years, the relapse rates diminish significantly. The authors hypothesized that AIs’ superiority over TAM was greater when patients had previously been treated with TAM. The reduction in breast cancer mortality with AIs compared to TAM appeared to be slight but persistent during years 0–4 and 5–9. This paper’s key findings consist mainly of the proportional risk reduction of approximately 30% in recurrence observed in the AIs vs. TAM comparison period and the proportional risk reduction of about 15% in mortality rate reported in the first 10 years. A 5-year course of TAM treatment can lower disease relapses by approximately half in years 0–4 and by approximately one-third in the following 5 years. Moreover, it decreases mortality rate by about 30% during the first decade and beyond (16). AIs administered for 5 years in the absence of any endocrine therapy lower relapse rates by about two-thirds while patients are actively on treatment and by about one-third in the following 5 years and reduce the mortality rate by approximately 40% throughout the first decade and perhaps beyond. Moreover, all the trials commencing endocrine treatment with an AI showed, collectively, a highly significant drop of 30% in recurrence during years 0 to 1, confirming the superiority of AIs over TAM (28). AIs are effective for lowering breast cancer relapses even in premenopausal women undergoing OFS (29). The literature details various relevant clinical trials comparing AIs with TAM in premenopausal women treated with OFS or ablation (30–33), and more recently, the EBCTCG published a meta-analysis to obtain a better definition of the risks and benefits of AIs vs. TAM in this patient setting (34). Trials for this paper were eligible if they randomly assigned premenopausal women affected by ER-positive operable breast cancer to receive an AI plus OFS vs. TAM plus OFS. ER-negative/PR-positive women were excluded from the data analysis because of uncertainties surrounding endocrine therapy’s efficacy in these patients. The primary outcomes were any recurrence (locoregional, distant, or contralateral metastasis from new primary breast cancer), breast cancer mortality, recurrence-free death, and all-cause mortality. The incidence and site of the second primary tumor and bone fractures were the secondary outcomes. The authors found that there was a decreased rate of recurrence for women receiving AIs compared to those receiving TAM, and the main gain was observed in years 0–4 with a significant drop in the relapse rate; a loss of benefits in years 5–9 was also observed. The 5-year absolute risk of relapse was 3.2% lower in the group of patients undergoing AIs with a similar absolute difference in 10-year relapse rates (14.7% in the AIs group and 17.5% in the TAM group). Distant recurrence appeared to reduce when AIs were given; however, at a median follow-up time of 8 years, the authors noted no significant difference in all-cause and breast cancer mortality. When the authors considered only trials for premenopausal women, few non-breast cancer deaths occurred (0.9% in the AIs group vs. 0.7% in the TAM group), most of which were due to a second primary tumor. Regarding secondary outcomes, the authors found that AIs caused a higher rate of fractures than TAM (6.4% vs. 5.1%). Regarding toxicity from AIs and TAM, each trial showed that they were similar for premenopausal and postmenopausal women; those receiving AIs showed higher osteoporosis rates, and those assigned to TAM suffered from endometrial abnormalities more frequently.

In summary, the results of this meta-analysis suggest that when AIs are initiated instead of TAM in premenopausal women, in addition to OFS, the absolute recurrence risk can be decreased by 3% at 5 and 10 years. In postmenopausal women, approximately 70% of all breast cancers are hormone receptor-responsive and candidates for endocrine therapy (35).

The benefits of endocrine therapy with AIs *versus* TAM in this patient setting have been solidly demonstrated in the literature (28), and other attempts to confirm these findings have led to the publication of different trials focusing on the superiority of AIs over TAM. The BIG 1-98 trial (18, 35–40) is a four-arm, phase III, double-blind, randomized trial comparing adjuvant letrozole *versus* TAM administered for 5 years in postmenopausal patients. This study was designed to describe these two drug sequences (2 years of one treatment followed by 3 years of the other). In 2011, the median follow-up (8.1 years) showed that letrozole administered as monotherapy provided a significant benefit regarding disease-free survival (DFS), overall survival (OS), distant recurrence-free interval (DRFI), and breast cancer-free interval (BCFI) compared with monotherapy with TAM (18). More recently, the BIG 1-98 long-term follow-up study (BIG 1-98 LTFU), which was an extension of the BIG 1-98 trial, provided an update on annual survival, disease status, and long-term adverse effects in women enrolled in this trial at a median follow-up time of 12.6 years (35). The primary endpoint was DFS; in contrast, the other endpoints included OS, DRFI, and invasive BCFI. The authors also examined breast cancer mortality and provided split analyses for monotherapy comparisons (letrozole vs. TAM) and sequential therapy comparisons *versus* TAM monotherapy. Consistent conclusions can be drawn regarding adjuvant AI therapy in postmenopausal patients and their long follow-up periods. The authors found that the DFS showed a relative risk reduction of 9% in patients receiving letrozole; the same was reported in a 10-year analysis of anastrozole in the ATAC trial (41). Other endpoints, compared with the previous analysis, showed slightly decreased hazard ratios (HRs) in favor of the letrozole arm, which could be due to non-breast events at such a higher median population age. Moreover, for the monotherapy comparison, 25.2% of patients receiving TAM selectively crossed over to letrozole, and 39.5% of patients receiving TAM selectively crossed over for the four-arm comparison. This event could have contributed to the improved outcome found in patients assigned to receive TAM and attenuated the benefits of letrozole. The authors even stated that they could not find any determining difference between the study arms when looking at myocardial infarction rates, osteoporosis or fracture, and cerebrovascular events at the long-term follow-up. However, they found a reduced thromboembolic event rate in the TAM group. In summary, this study demonstrated that initial letrozole can offer continued and slightly attenuated benefits compared with TAM in postmenopausal women with hormone-responsive early breast cancer (35). Regarding patients with locally advanced and metastatic endocrine-responsive postmenopausal breast cancer, a meta-analysis of phase III randomized controlled trials comparing first-line endocrine therapy with third-generation AIs and TAM was reported in the literature. This study examined OS and addressed whether the progression-free survival (PFS) benefit of AI therapy results from a reduction in *de novo* resistance or a delay in acquired resistance to endocrine therapy. It included data from four large trials, all designed with TAM administered in the control arm and different AIs (exemestane, anastrozole, and letrozole) administered in the experimental arm. Every trial was reviewed for the clinical benefit rate, duration of clinical benefit (DoCB), PFS, and OS. The authors observed that AIs enabled more patients to achieve clinical benefits (CB) than TAM. The DoCB appeared slightly higher for AIs but did not significantly differ from TAM. In contrast, the PFS was statistically significantly different between the two groups in favor of AIs. Finally, even after excluding letrozole from the data, OS did not significantly differ between the two arms. The study concludes that, in the first-line setting, the choice of an AI instead of TAM has a significant clinical benefit as it increases the duration of tumor control by prolonging the PFS (12).

## 4 To extend or not to extend

Despite the widely demonstrated success of adjuvant endocrine therapy given for 5 years, it is also recognized that hormone-responsive breast cancer can be linked to a prolonged recurrence risk after the first 5 years of therapy (42). More than 50% of relapses occur after the first 5 years of treatment (43). Recently, many clinical trials have focused on evaluating the optimal duration and efficacy of endocrine therapy (44–52). It has been demonstrated that 10 years of adjuvant TAM can lower both recurrence risk and mortality in patients with breast cancer over time, thanks to its major impact between the 9th and 10th years of follow-up (53, 54). However, there has been extensive debate in the scientific community regarding using AIs as an extended therapy, and their optimal duration has not yet been established (55, 56). Clinical trials in recent years led to the publication of conflicting data and non-definitive conclusions. Specifically, trials such as MA.17, MA.17R, and 6a demonstrated a DFS benefit (45, 56, 57), with a particular improvement in OS in the MA.17 trial, in patients diagnosed with node-positive diseases and in those previously treated with TAM for 5 years. However, these results were not fully confirmed by the latest published randomized trials (47, 49, 51). Among these studies, only MA.17 was able to show that both DFS and OS improved in a statistically significant manner, especially in high-risk patients (those with larger tumors and/or positive nodes). The efficacy and toxicity of extended AI therapy in patients with hormone receptor-positive breast cancer have also been investigated in two subsequently published meta-analyses (58, 59). The first analysis (58) included eight studies (45, 47–51, 56, 57) in which endocrine therapy was employed: letrozole was investigated in four studies, anastrozole in three, and exemestane in one. Most of these studies evaluated the effects of extending AI therapy for up to 5 years (**Tables 1**, **2**). The authors found improved DFS at a median follow-up time of 64.1 months, and major benefits were observed in patients with positive nodes. In addition to this increase in DFS rates, extending the therapy with AIs did not show any benefit regarding OS. This meta-analysis revealed a general increase of 22% in DFS, and this benefit appeared to be greater in patients with node positivity. The authors’ statement seems to be in accordance with the MA.17 results and with the DATA trial (49) *post-hoc* subgroup analysis, which again demonstrated that patients with node positivity benefit from an extension of AI therapy. Moreover, although the authors failed to find a significant improvement in OS, all trials included in the study showed a significant decrease in recurrence risk, a benefit in the breast cancer-free interval, and a 28% reduction in the cumulative risk of developing distant relapses (58). The second meta-analysis (59) included eight clinical trials (47, 49, 51, 52, 56, 57, 60, 61), most of which were the same as those previously discussed but focused on local and distant recurrence, contralateral tumors, non-breast cancer-related death, and toxicity. Most studies have evaluated the effects of extending AI therapy in patients who have already completed 5-year endocrine AI regimens. The data on DFS and OS were the same as those used in a previous meta-analysis. The pooled analysis demonstrated that the risk of death from any cause and non-breast cancer-related causes did not decrease when the AI was extended. Local and distant recurrences did not improve significantly. With regard to toxicity, the study reported an increased rate of osteoporosis, bone pain, bone fractures, hypertension, cardiovascular events, arthralgia, and myalgia, which are recognized as typical of this drug class. The authors of this meta-analysis concluded that extended AI therapy in postmenopausal women can provide better outcomes for recurrence rate; in contrast, the toxicity rates suggest that it should be chosen based on patient and disease characteristics. Moreover, owing to the carryover effect of endocrine therapy, which produces an absolute survival benefit that increases over time and becomes significant, especially after 10 years, it is clear that the follow-up period should be adequate to evaluate the real benefit of extended adjuvant endocrine therapy (59). Another matter of debate is the optimal duration of extended endocrine therapy for breast cancer. A meta-analysis (42) examined the optimal duration of extended AI therapy in postmenopausal patients who had already completed their 5-year regimens. The authors classified the included studies according to the total endocrine therapy extension time, assigning nine randomized controlled trials to three classes. The first class grouped the patients according to 10 years vs. 5 years of therapy (AERAS, MA.17, NSABP B-42, and NSABP B-33). The second group included studies comparing 7 to 8 years and 5 years of treatment (ABCSG 6a, DATA, and GIM4 LEAD). The last class grouped trials examined 10 years vs. 7–8 years of therapy (ABCSG-16 and IDEAL). This central meta-analysis demonstrated that DFS improved when endocrine therapy was extended, especially when the extension time frames were 5–10 and 5 –7 to 8 years. However, when prolonging therapy from 7 to 8 years to 10 years, the DFS did not improve. Regarding OS, the results showed that extended therapy was not associated with a lower risk of death from any cause, regardless of the time frame of the extension. When drugs are prolonged from 5 to 10 years, the risk of bone fracture, osteopenia/osteoporosis, bone pain, myalgia, joint stiffness, and alopecia increases. The following conclusions can be drawn from the subgroup analysis. Patients affected by node-positive, hormone receptor-positive, or tumors > 2 cm in size treated only with TAM or sequential TAM-AIs for 5 years may significantly benefit from the extension of endocrine therapy as soon as they are at a higher risk of recurrence (62). The extension of AI therapy in such patients for 2 years, rather than 5 years, seems to be the right choice to maximize the benefits without increasing the toxicity (63).

**Table 1:** DFS in studies of extended vs. standard endocrine therapy (sub-group analysis).

	10 years vs. 5 years			7–8 years vs. 5 years			10 years vs. 7–8 years		
	Study	HR (95% CI)	Pooled HR (95% CI)	Study	HR (95% CI)	Pooled HR (95% CI)	Study	HR (95% CI)	Pooled HR (95% CI)
5 years of TAM	MA17	0.58 (0.45, 0.76)	0.61 (0.49, 0.76)	ABCSG6a	0.40 (0.22, 0.73)	0.40 (0.22, 0.73)	ABCSG-16	0.99 (0.82, 1.20)	1.00 (0.83, 1.20)
	NSABP B-33	0.68 (0.45, 1.03)					IDEAL	1.06 (0.50, 2.25)	
2–3 years of TAM and then 3–2 years of AIs	AERAS	0.60 (0.16, 1.99)	0.74 (0.57, 0.97)*	ABCSG6a	1.13 (0.56, 2.25)	0.82 (0.71, 0.95)*	ABCSG-16	1.06 (0.84, 1.34)	1.02 (0.85, 1.23)*
	NSABP B-42	0.75 (0.57, 0.99)		DATA	0.79 (0.62, 1.02)		IDEAL	0.97 (0.73, 1.30)	
				GIM4 LEAD	0.82 (0.68, 0.98)				
5 years of AIs	AERAS	0.55 (0.39, 0.78)	0.72 (0.44, 1.18)^a^,*				ABCSG-16	0.86 (0.52, 1.41)	0.82 (0.60, 1.13)
	NSABP B-42	0.91 (0.75, 1.10)					IDEAL	0.80 (0.53, 1.21)	

Chen J, Breast Cancer (2021) 28:630–643.

DFS, disease-free survival; TAM, tamoxifen; AI, aromatase inhibitor; HR, hazard ratio; CI, confidence interval.

**Table 2:** OS in studies of extended vs. standard endocrine therapy.

10 years vs. 5 years		7–8 years vs. 5 years		10 years vs. 7–8 years	
Study	HR (95% CI)	Study	HR (95% CI)	Study	HR (95% CI)
AERAS	1.39 (0.33, 5.80)	ABCSG6a	0.89 (0.59, 1.34)	ABCSG-16	1.01 (0.82, 1.23)
MA17	0.82 (0.57, 1.19)	DATA	0.91 (0.65, 1.29)	IDEAL	1.04 (0.78, 1.38)
NSABP B-42	1.15 (0.87, 1.27)				

Chen J, Breast Cancer (2021) 28:630–643.

DFS, disease-free survival; OS, overall survival; HR, hazard ratio; CI, confidence interval; TAM, tamoxifen; AIs, aromatase inhibitors.

## 5 AIs and CDK4/6 inhibitor combinations in metastatic and early breast cancer

Cancer cells frequently present with cell cycle abnormalities, considered potential therapeutic targets. Cyclin-dependent kinases (CDKs) are cell cycle transition and cell division-governing regulatory enzymes that involve the tumor suppressor retinoblastoma (Rb) protein as a regulator of cellular proliferation. The CDK4/6-Rb pathway is often present in ER-positive breast cancer, as estrogens promote the evolution from the G1 to the S phase. This pathway is considered a key mediator of endocrine resistance (64). The binding mechanism between estrogen and its alpha receptor promotes cyclin D1 transcription, CDK4/6 activation, and Rb phosphorylation, immediately leading to cell cycling. Targeting and inhibiting CDK4/6 causes the cell to stop its cycle in the G1 phase, resulting in lower cell viability (65). Palbociclib, ribociclib, and abemaciclib represent the current armamentarium for developing highly selective oral CDK4/6 inhibitors. Various clinical trials performed in both metastatic breast cancer (mBC) and early breast cancer (eBC) patient settings (66–81) have extensively investigated these drugs, showing an impressive impact on outcomes (82). The combined use of CDK4/6 inhibitors and adjuvant endocrine therapy represents the most significant advancement in managing both advanced mBC and eBC, and its development has dramatically changed the therapeutic scenario for this disease. Patients can be exposed to this class of drugs for a long time, and the median PFS is approximately 28 months. Drug selection is an important aspect in this patient setting (83). The literature includes systematic reviews and meta-analyses that explore and compare the toxicity and tolerability profiles of palbociclib, ribociclib, and abemaciclib toxicity and tolerability profiles (83–85). When choosing one drug over another in everyday clinical practice, the right decision should be tailored to every patient and driven by comparative toxicity studies. A recent meta-analysis published in 2020 compared ribociclib and abemaciclib to palbociclib and quantified the treatment-related side effects for each endocrine therapy regimen (AIs or fulvestrant). The analysis revealed that ribociclib presented higher grade 3 to 4 nausea and vomiting than palbociclib but a non-significant lower grade 3 to 4 hematological toxicity, independent of the underlying endocrine therapy. Abemaciclib demonstrated higher grade 1v2 and 3 to 4 diarrhea and grade 1 to 2 nausea and vomiting, with lower grade 3 to 4 neutropenia than palbociclib. When combined with AIs, abemaciclib resulted in a lower percentage of grade 3–4 fatigue than palbociclib. Treatment discontinuation was similar for palbociclib and ribociclib; however, the rate was higher in patients who received abemaciclib. Moreover, the association rate between grade 3 to 4 diarrhea and abemaciclib and palbociclib was higher in patients receiving fulvestrant than in those receiving AIs. The authors stated that the differences in inhibitor-specific toxicity could be explained by the pharmacological features of the three drugs. Abemaciclib had the highest activity against CDK4 and CDK6; in contrast, palbociclib and ribociclib had similar potencies against CDK4 and CDK6. However, ribociclib is usually associated with higher levels of unbound blood. Abemaciclib more specifically binds CDK4; on the other hand, palbociclib has an equivalent specificity. If CDK6 appears to be the predominant CDK in hematopoietic cells, abemaciclib may cause a lower rate of neutropenia because of its favorable hematopoietic inhibition. Moreover, its action against CDK7 and CDK4 may partially explain its increased intestinal toxicity.

The authors conclude that it is likely that the “modulable” inhibition of CDK4 and CDK6 in the three drugs is more associated with their toxicity than their efficacy (83). Little is known about AI-induced musculoskeletal symptoms (AIMSS), one of the most detrimental adverse effects experienced by patients receiving endocrine therapy. In a recent review, the authors summarized all available data on this adverse effect analyzed in randomized phase III trials evaluating AI monotherapy or combined with CDK4/6 inhibitors in patients with metastasis. They focused on the additive influence of CDK4/6 inhibitors on AIMSS and found that the incidence of arthralgia decreased in patients who received combined therapy. Myalgia was also reduced by the addition of CDK4/6 inhibitors, which was consistent with the results observed for arthralgia. Regarding bone pain, the combination therapy resulted in a 2.9%–8.5% rate of incidence compared to 7%–32.9% reported for patients receiving AIs as monotherapy. Palbociclib presented with bone pain more commonly than ribociclib or abemaciclib. Overall, patients with metastases receiving CDK4/6 inhibitors had fewer adverse events than those receiving AI monotherapy. The authors also examined the back pain rate and observed that it manifested mostly in patients treated with AI monotherapy compared to those receiving combination therapy. Data from this review suggest that combining CDK4/6 inhibitors with a pre-existing AI therapy regimen tends to lessen the incidence of AIMSS; however, more data, both in adjuvant and metastatic settings, are required to clarify these findings (85). The literature demonstrates that besides the proven efficacy of the association between AIs and CDK4/6 inhibitors, these drugs have a safe toxicity profile that allows their wide use in patients with eBC and mBC. The literature can support everyday clinical practice and the need to choose one drug over another; however, further data to support the day-to-day decision-making processes is required.

## 6 Old problems, new problems

As indicated above, AIs are a cornerstone for both pre- and postmenopausal women with hormone receptor-positive breast cancers. During adjuvant endocrine therapy, no significant decrease in health-related quality of life has been reported in several large-scale trials (86–88). Despite these data, early disruption is frequent in different research and clinical practice settings, ranging from 30% to 70% (89). The reasons for the discontinuation of endocrine therapy are usually multifactorial. The data suggest that up to 30% of patients do not adhere to AI therapy because of its adverse effects. Multifactorial barriers hampering correct adherence are usually associated with patient-, physician-, medication-, and system-related variables, and all of these factors are usually variously combined in women with a growing number of breast cancer survivors. Adherence to endocrine therapy is fundamental, as it can improve patient outcomes and survival curves (89, 90). Patients who disrupt their AI treatment early are exposed to an increased risk of all-cause mortality, cancer death, and recurrence because they do not fully receive the intended therapy-related benefits (91). Most women who fail to adhere to their endocrine treatment discontinue therapy during the first 6 months because the most severe toxicities related to AIs tend to occur within this period (92–94) (**Table 3**).

**Table 3:** Common adverse events in patients undergoing therapy with aromatase inhibitors in an extended and non-extended setting and their incidence pooled risk ratio.

	10 years vs. 5 years			7–8 years vs. 5 years			10 years vs. 7–8 years		
	Study	RR (95% CI)	Pooled RR (95% CI)	Study	RR (95% CI)	Pooled RR (95% CI)	Study	RR (95% CI)	Pooled RR (95% CI)
Bone fracture	AERAS	2.44 (1.13, 5.28)	1.27 (1.05, 1.54)*	DATA	1.33 (0.97, 1.81)	1.36 (1.01, 1.84)*	ABCS 16	1.49 (1.10, 2.00)	1.57 (1.22, 2.02)*
	MA17	1.15 (0.91, 1.47)		GIM4 LEAD	1.81 (0.61, 5.37)		IDEAL	1.79 (1.11, 2.90)	
	NSABP B-42	1.30 (0.81, 2.11)							
	NSABP B-33	1.39 (0.79, 2.45)							
Osteopenia/osteoporosis	AERAS	1.18 (1.01, 1.37)	1.24 (1.10, 1.40)*	GIM4 LEAD	1.73 (1.22, 2.45)	1.73 (1.22, 2.45)*	IDEAL	1.70 (1.28, 2.26)	1.70 (1.28, 2.26)*
	MA17	1.35 (1.11, 1.65)							
	NSABP B-42	0.50 (0.12, 1.99)							
Bone pain (including arthralgia)	AERAS	1.63 (1.28, 2.07)	1.28 (1.11, 1.49)^a^,*	ABCSG 6a	1.34 (1.03, 1.73)	1.34 (1.03, 1.73)*	IDEAL	1.11 (0.88, 1.40)	1.11 (0.88, 1.40)
	MA17	1.23 (1.11, 1.36)							
	NSABP B-42	1.15 (1.00, 1.32)							
	NSABP B-33	1.99 (0.60, 6.58)							
Joint stiffness	AERAS	2.42 (1.68, 3.49)	2.40 (1.68, 3.43)*				IDEAL	0.94 (0.66, 1.35)	0.94 (0.66, 1.35)
	MA17	1.99 (0.37, 10.86)							
Myalgia	MA17	1.23 (1.07, 1.41)	1.24 (1.10, 1.39)*						
	NSABP B-42	1.26 (1.00, 1.59)							
Alopecia	MA17	1.42 (1.09, 1.85)	1.42 (1.09, 1.85)*				IDEAL	0.91 (0.64, 1.30)	0.91 (0.64, 1.30)
Depression	MA17	1.09 (0.87, 1.38)	1.12 (0.89, 1.39)				IDEAL	0.82 (0.56, 1.19)	0.82 (0.56, 1.19)
	NSABP B-42	1.39 (0.62, 3.13)							
Hypertension	MA17	1.01 (0.80, 1.28)	1.00 (0.81, 1.24)						
	NSABP B-42	0.96 (0.57, 1.61)							

RR, risk ratio; CI, confidence interval.

aDue to the heterogeneity between studies, this pooled value was from the random-effects analysis, while others were from the fixed-effect analysis.

*P < 0.05.

Concerning endocrine therapy, one of the most significant adverse effects that play a fundamental role in the quality of life of this group of patients is bone loss. As soon as the fracture risk increases, a practical approach is mandatory to avoid worsening bone quality and quantity, which leads to fragility. Endocrine therapy works by directly or indirectly removing the effect of estradiol on the breast tissue. The same effect is exerted on the bone tissue, leading to bone loss (95, 96). A position paper published in 2017 by seven international bone and cancer societies thoroughly examined AI-induced bone loss (AIBL) (97). This produced an algorithm for AIBL management based on clinical risk factors and bone mineral density (BMD). More recently, interesting meta-analyses on bone health in patients treated with endocrine therapy clarified the state of the art of the issue. In a review by Waqas et al. (98), data published in 2017 on the relationship between AI therapy and bone health were analyzed. The authors included novel fracture risk assessment tools, such as the trabecular bone score and vertebral fracture assessment (VFA), to update the clinical management strategy defined in a position paper published in 2017. They even attempted to address bone loss and fracture risk in premenopausal women. The authors suggest that all women receiving AIs and/or OFS should undergo dual-energy X-ray absorptiometry scan (DEXA) and biochemical testing with a meticulous medical history and physical examination to rule out further secondary factors capable of causing osteoporosis. VFA, together with DEXA, could be part of the screening or follow-up for all postmenopausal and osteopenic premenopausal patients on endocrine therapy. The authors even advise against using the conventional fracture risk assessment tool, FRAX, as its role is still not well established and validated in women under 40, and it can underestimate the fracture risk. They stated that physicians should stress the role of physical exercise in women undergoing endocrine therapy, along with an adequate intake of calcium and vitamin D. According to the current ESMO guidelines, every woman starting AIs and/or OFS should undergo a fracture risk assessment using conventional risk factors and BMD measurement with DEXA and VFA. All women presenting a BMD score of 2.0 SD or more than two risk factors (prior fragility fractures, parental history of hip fracture, diabetes, BMI 20 kg/m^2^, rheumatoid arthritis, recurrent falls, use of glucocorticoids for more than 3 months, current smoking, and alcohol consumption) should be offered anti-resorptive therapy. Regarding follow-up, when no antiresorptive therapy has started, DEXA should be repeated yearly after AI initiation and every 2 years in patients on antiresorptive medication. The recommended antiresorptive therapy extension should be based on the extension of endocrine therapy and specific absolute fracture risk. When extended adjuvant endocrine therapy is considered, it may be necessary to consider whether or not to start anti-resorptive drugs based on an overall evaluation of individualized risk-to-benefit ratios and potential side effects. It can be affirmed that as breast cancer develops into a chronic disease, bone loss and fracture risk in patients receiving endocrine therapy will require special attention. Cardiotoxicity is a major concern in the prescription of AIs. Several adjuvant randomized controlled trials comparing AIs with TAM have shown that the risk of developing a cardiovascular disease increases with AIs (99–101). In contrast, previous clinical studies have indicated a favorable cardiovascular effect of TAM, specifically in reducing cholesterol and low-density lipoprotein blood levels and increasing high-density lipoprotein (101–106). Therefore, the AI-associated increased risk of cardiovascular events could be interpreted in light of the cardioprotective effects of TAM. An interesting systematic review and meta-analysis was published in 2017 to determine whether AI therapy is associated with a higher risk of cardiovascular events. This study included 19 randomized clinical trials. The authors stated that, given the fact that TAM is associated with a 33% decrease in the risk of cardiovascular events in all trials, including those comparing TAM to placebo or no treatment, it could be concluded that the cardioprotective effect of TAM explains the increased risk observed in randomized controlled trials comparing AIs to TAM. MA.17 and MA.17R supported this finding in an extended adjuvant setting; the authors declared no association between AIs and cardiovascular events or ischemic heart disease (45, 56).

## 7 Drug adherence: new approaches to connected solutions

In recent years, several attempts have been made to enhance adherent patient behavior regarding endocrine treatments. Constantly improving and using user-friendly technology can certainly help patients and physicians maintain contact with each other. Moreover, because it is easy to use and widespread, a technology that enables a quick response could be helpful for clinicians in ameliorating symptoms and obtaining fast answers.

Basic and advanced technologies have been used in studies and trials designed to improve adherence to endocrine therapy.

In 2018, Graetz et al. (107) conducted a pilot randomized controlled trial to analyze the use, feasibility, and short-term effects of a web-based application specifically released for patients with breast cancer.

The app communicated the onset of adverse symptoms and AI adherence through built-in alerts sent to the patient’s care team. Using their own enabled devices (smartphones and computers), patients can share real-time health information with their team of oncologists. This study found that this app, which provides reminders for real-time reporting of AI adherence and treatment-related side effects, was feasible and improved short-term adherence. The use of the app decreased when it worked without weekly reminders, and this finding was particularly frequent among black participants, younger women, and those with lower literacy levels. Adherence interventions are necessary for this sector of the population. Moreover, in patients using the app with weekly reminders, the authors reported a smaller increase in symptom burden, suggesting that they may have received adequate management due to the app-based real-time reports. Finally, from the clinicians’ point of view, the staff reported that participating in the initiative had a minimal impact on the daily workflow, suggesting that this patient-centered way of working could be widely implemented without heavily impacting the everyday burden of clinical work.

Two other randomized controlled trials (108, 109) investigated the feasibility of employing simple text messaging technology to limit the early discontinuation of breast cancer endocrine therapy. The first study found that a biweekly unidirectional message sent to patients undergoing endocrine treatment for 36 months did not affect adherence compared with usual care. This finding was perhaps because the project did not actively engage patients and was insufficient to produce a behavioral change (108).

Another randomized trial used a mobile phone text message reminder system (SMS) to achieve two primary objectives: to evaluate whether SMS ameliorates drug adherence compared to standard care in patients undergoing AI therapy and to assess whether SMS actively affects the blood levels of androstenedione, estradiol, and estrone. The authors found that, at 6 months, the percentage of adherence in the SMS group was higher (72,4%) than that in the standard care group (59.5%); however, at 1 year, the value for the SMS group was not significantly higher than that in the standard care group (68.9% and 65.8%, respectively). Moreover, they observed that the androstenedione blood level measured at 1 year was comparable in both groups, and the estrone level was higher in the SMS group; however, this difference was not statistically significant. Estradiol levels were not significantly lower in the SMS group than in the standard care group. They concluded that even though the SMS reminder system appeared to have high acceptability among patients, it showed only a significant short-term effect in improving medication adherence (109).

Further research needs to be conducted on this topic, focusing on how technology can help clinicians and patients manage their adherence to endocrine therapy, thereby paving the way for better and longer therapeutic responses. On the other side, it is important to prevent endocrine overtreatment especially in older patients. An interesting genomic approach has been reported to select the ultralow-risk 70-gene signature patients as candidates for treatment de-escalation (110).

## 8 Conclusions

Currently, AIs are the cornerstone in the management of breast cancer in every patient setting (pre- and postmenopausal, eBC, and mBC). Their use is widespread, as the literature has fully demonstrated their efficacy in improving survival and their low toxicity profile. Adverse effects on bone health, the cardiovascular system, and metabolism can be easily handled with dedicated network paths in which patients feel completely managed by experts and are globally followed up on every aspect of their disease. Greater efforts should be made to improve adherence to endocrine therapy, especially in a selected subset of patients undergoing extended adjuvant therapy because of the high risk of relapse after 5 years. Modern technology can help physicians; however, reaching the optimal point remains a long way off. The more patients follow endocrine therapy, the more we can achieve longer and better responses.

## Author contributions

Conceptualization, DG; writing, original draft preparation, Ethos srl.; writing, review, and editing, Ethos srl; supervision, DG. All the authors have read, reviewed, and agreed to the published version of the manuscript.
