# Corrigendum: Aromatase inhibitors: the journey from the state of the art to clinical open questions

**DOI:** 10.3389/fonc.2024.1369562

**Published:** 2024-01-23

**Authors:** Daniele Generali, Rossana Berardi, Michele Caruso, Marina Cazzaniga, Ornella Garrone, Ida Minchella, Ida Paris, Carmine Pinto, Sabino De Placido

**Affiliations:** ^1^ Breast Cancer Unit, Azienda Socio Sanitaria Territoriale di Cremona, Cremona, Italy; ^2^ Department of Medical, Surgery and Health Sciences, University of Trieste, Trieste, Italy; ^3^ Medical Oncology, Azienda Ospedaliera Universitaria (AOU) delle Marche, University Politecnica delle Marche, Ancona, Italy; ^4^ Humanitas Istituto Clinico Catanese, Breast Centre Humanitas Catania, Catania, Italy; ^5^ School of Medicine and Surgery University of Milano Bicocca, Milan, Italy; ^6^ Phase 1 Research Unit, Azienda Socio Sanitaria Territoriale (ASST) Monza, Monza, Italy; ^7^ Medical Oncology, Fondazione IRCCS Ca’ Granda Ospedale Maggiore Policlinico, Milan, Italy; ^8^ Division of Early Drug Development, European Institute of Oncology IRCCS, Milan, Italy; ^9^ Department of Woman and Child Health and Public Health, Division of Gynecologic Oncology, Fondazione Policlinico Universitario Agostino Gemelli Istituto di Ricerca e Cura a Carattere Scientifico (IRCCS), Rome, Italy; ^10^ Medical Oncology Unit, Comprehensive Cancer Centre, Azienda Unità Sanitaria Locale - Istituto di Ricerca e Cura a Carattere Scientifico (AUSL-IRCCS) di Reggio Emilia, Reggio Emilia, Italy; ^11^ Department of Clinical Medicine and Surgery, University of Naples Federico II, Naples, Italy

**Keywords:** aromatase inhibitors, bone loss, cardiotoxicity, drug adherence, breast cancer

In the published article, there was an error in affiliation 8. Instead of “Division of Early Drug Development, European Institute of Oncology, Milan, Italy”, it should be “Division of Early Drug Development, European Institute of Oncology IRCCS, Milan, Italy”.

The authors apologize for this error and state that this does not change the scientific conclusions of the article in any way. The original article has been updated.

